# Climate change and the burden of healthcare financing in African households

**DOI:** 10.4102/phcfm.v15i1.3743

**Published:** 2023-01-31

**Authors:** Chinwe F. Ezeruigbo, Abel Ezeoha

**Affiliations:** 1 Department of Nursing, Faculty of Health Sciences and Technology, Ebonyi State University, Abakaliki, Nigeria; 2 Department of Banking and Finance, Faculty of Management Sciences, Alex-Ekwueme Federal University, Abakaliki, Nigeria

**Keywords:** climate change, out-of-pocket health expenditure, household income, primary health care, Africa

## Abstract

Climate change is a mounting pressure on private health financing in Africa – directly because of increased disease prevalence and indirectly because of its negative impact on household income. The sources and consequences of the pressure constitute an important area of policy discourse, especially as it relates to issues of poverty and inequality. Relying on a panel dataset involving 49 African countries and the period 2000–2019, as well as a random effect regression analysis, this report shows that climate change has a positive and significant impact on the level of out-of-pocket health expenditure (OPHE) in Africa, and an increase in the level of greenhouse (CO_2_) emissions by 1% could bring about a 0.423% increase in the level of OPHE. Indirectly, the results show that, compared with the regional average, countries that have higher government health expenditure levels, above 1.7% regional average, and face higher climate change risk may likely record an increase in OPHE. Alternatively, countries with higher per capita income (above the regional annual average of $2300.00) are likely to record a drop in OPHE. Countries with lower climate change risk and a lower than the regional average age dependency (above the regional average of 80.4%) are also likely to record a drop in OPHE. It follows that there is a need for policy alignment, especially with regard to how climate change influences primary health care funding models in Africa.

**Contribution:**

The results of this research offer policymakers in-depth knowledge of how climate change erodes healthcare financing capacity of government and shifts the burden to households. This raises concerns on the quality of accessible healthcare and the link with poverty and inequality.

## Introduction

Climate change is confirmed to be among the greatest problems currently threatening human health. This is especially so in Africa, where a greater percentage of the population is poor and where public health systems are under severe pressure because of fragile socio-economic conditions and climate change.^1^ A 2019 report by the World Meteorological Organization, for instance, identified that ‘warmer temperatures and higher rainfall are responsible for increasing the transmission of vector-borne diseases such as dengue fever, malaria and yellow fever’ in Africa.^2^ In the region, studies have also linked the problems of poverty, inequality, food insecurity, internal displacement because of conflicts and drought to the increased risk of climate change.^3^

**FIGURE 1:**
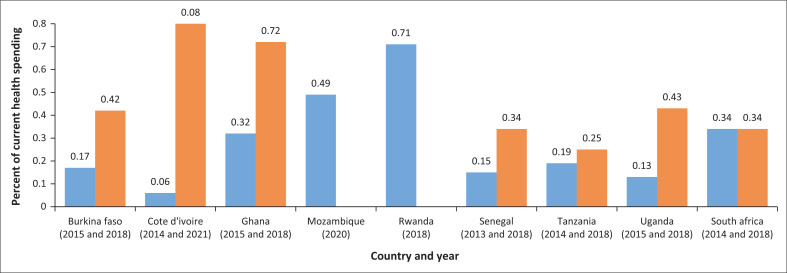
Government primary health care spending as % of current health spending.

Induced largely by climate change, the deteriorating health situation in Africa has therefore mounted enormous pressure on the demand for primary health care (PHC).^4^ The situation is worsened by the fact that the majority of the affected population comprises individuals whose access to health care is already constrained by shortages in health personnel and health facilities.^5^ In most of the countries, public health financing falls short of the African Union’s prescribed 15% budgetary allocation.^6^ As shown in Figure 1, despite little improvement over the years, none in the region has been able to meet up with the World Health Organization (WHO) requirement that ‘every country allocate or reallocate an additional 1% of gross domestic product (GDP) to PHC from government and external funding sources’.^7^

The focus on PHC is important because the subsector is recognised by the WHO as potentially the most inclusive, equitable and cost-effective way of attaining the goal of universal health coverage in developing countries.^8,9^ How the subsector is funded also has a direct bearing on inequality and poverty. The WHO, for instance, recorded that ‘about 930 million people worldwide are at risk of falling into poverty because of out-of-pocket health expenditure (OPHE) of 10% or more of their household budget’.^7^ It has also been established that for the 5.6 billion people in low-to middle-income countries, ‘half of all health expenditure is through out-of-pocket payments’.^9^ Amidst the high poverty rate, OPHE has become a norm in almost all countries – thus widening inequality and exacerbating public health concerns in the region.^10^

Unfortunately, while there is evidence linking climate change to worsening health status, very few studies have attempted to examine this relationship from the perspective of the financial burden of PHC delivery. This short report contributes to the current debate by attempting to address pertinent questions relating to the following:

- To what extent do climate change risks directly shift the burden of healthcare financing to individuals and households vis-à-vis an increase in OPHE?
- How does climate change interact with income level, government health expenditure and age dependency to influence out-of-pocket spending on healthcare in Africa?

**FIGURE 2:**
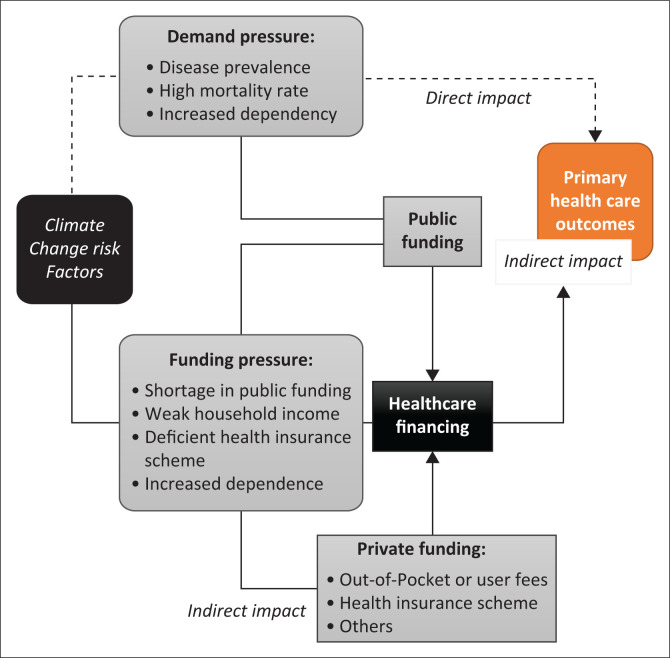
Climate change–health financing model.

As shown in Figure 2, the theoretical link between climate change and healthcare financing is complex, as it can be both direct and indirect. This report examines both the direct and the indirect impacts using the lens of out-of-point health financing.

## Methods

The data for this report, which involved a panel of 49 African countries and the period 2000–2019, were sourced from both the World Development Report and the data stream of the Primary Health Care Performance Initiative (PHCPI).^11^ The observed main variable of interest is OPHE, defined as the ‘share of out-of-pocket payments of total current health expenditures. OPHEs are spending on health directly out-of-pocket by households’.^12^ The major predictor variable is climate change. The control variables, based on their theoretical relevance, are the share of public health expenditure, income level, infant mortality rate and age dependency rate. The variables were converted to their respective natural logarithms.

In terms of estimating the direct and indirect impact of climate change on the burden of PHC financing in African households, the random effect panel regression was applied. This was informed by the relatively small number of observations involved in the dataset.

### Ethical considerations

The study leading to this short report adhered to all the required ethical standards. All the data used and the materials cited are in the public domain. There was no direct contact with human or animal subjects.

## Results

Table 1 contains the descriptive results of the report. For the 49 African countries selected, the average OPHE is estimated at 40.8% – made up of a range of 3% (for Namibia) and as high as 84.2% (for Comoros). This means that on average, OPHE in Africa falls within ‘[c]atastrophic expenditure’ – defined by the WHO ‘as out-of-pocket health payments exceeding 40% of household non-subsistence spending’. The mean value of climate change (measured by CO_2_ emissions in kg per capita per year) is 0.172, with a range of 0.024 for the Democratic Republic of the Congo to 0.799 for South Africa.

**TABLE 1:** Descriptive results.

Variables	Mean	s.d.	Minimum	Maximum
Out-of-pocket expenditure (percentage of current health expenditure)	40.8	20.3	3.0	84.2
CO_2_ emissions (kg per capita)	0.2	0.1	0.02	0.8
Mortality rate, infant (per 1000 live births)	56.0	25.9	9.9	138.1
Age dependency ratio (% of working-age population)	80.4	17.8	7.5	111.9
Domestic general government health expenditure (percentage of GDP)	1.7	1.2	0.1	6.0
Per capita income (US$)	2299.7	3173.9	8.1	22942.6

CO_2_, level of greenhouse emission GDP, gross domestic product

Table 2 reports the results of the empirical analysis. It confirms that climate change has a direct negative and significant impact on the level of OPHE in Africa. Specifically, it shows that an increase in the level of greenhouse (CO_2_) emissions by 1% brings about a 0.423% increase in the level of OPHE. Among the predictor variables, infant mortality significantly increases OPHE, whereas increase in government health expenditure and per capita income significantly lower OPHE. Indirectly, the results show that the effect of climate change on OPHE is sensitive to the level of government health expenditure, national income level and age dependency. This means that, comparative to the regional average, how climate change impacts on OPHE depends on whether the level of government health expenditure is high or low, whether the income level is high or low and whether the prevailing age dependency ratio is high or low.

**TABLE 2:** Regression results on the impact of climate change on OPHE.

Variable	1. Direct effect		2. Interactive effect	

	*p*-value	s.e.	*p*-value	s.e.
Climate change	0.423‡	0.197	2.663†	0.637
Infant mortality	0.240†	0.044	0.150‡	0.073
Age dependency	0.041	0.047	0.088	0.107
Government health expenditure	−0.214†	0.018	−0.305†	0.106
Per capita income	−0.087†	0.021	0.075	1.119
Climate change: Government health expenditure	-	-	0.091	0.528
Climate change: Per capita income	-	-	−1.668†	0.649
Climate change: Age dependency	-	-	−1.969†	0.716
Constant	1.317†	0.106	1.382†	0.103
*R* ^2^	0.229	-	0.318	-
Wald X2	381.17†	-	73.27†	-
Number of observations	979	-	979	-

Note: The direct equation is estimated using the natural log values of all the variables, which makes the coefficient interpretable in percentage (%) terms.

OPHE, out-of-pocket health expenditure.

† , represents prob ≤ 0.01;

‡ , represents prob ≤ 0.05.

The evidence suggests also that countries that have higher government health expenditure levels above the 1.7% regional average and face higher climate change risk may likely record an increase in OPHE, which confirms the overwhelming pressure the former can have on primary health outcomes.

## Conclusion and implication

This report indicates that indeed climate change might have significantly shifted the burden of PHC financing to individuals and households in Africa. Specifically, it is shown that climate change (measured as the degree of greenhouse emission) has both direct and indirect effects on OPHE. Directly, it brings about an increase in OPHE vis-à-vis the demand pressure on PHC services. Indirectly, climate change appears to be frustrating governments’ attempts to increase funding for the health sector. The implication of the results is also that only individuals and households with income levels above the poverty level are in a position to cope with the climate change–induced pressure on OPHE. It follows that there is need for policy alignment, especially with regard to how climate change influences PHC funding models in Africa.
